# Two-Dimensional Omnidirectional Wind Energy Harvester with a Cylindrical Piezoelectric Composite Cantilever

**DOI:** 10.3390/mi14010127

**Published:** 2023-01-03

**Authors:** Mingyong Xin, Xueling Jiang, Changbao Xu, Jing Yang, Caijiang Lu

**Affiliations:** 1 Electric Power Research Institute of Guizhou Power Grid Co., Ltd., Guiyang 550002, China; 2 School of Mechanical Engineering, Southwest Jiaotong University, Chengdu 610031, China

**Keywords:** wind energy harvester, two-dimensional, cylindrical beam, piezoelectric tube

## Abstract

To improve the response-ability of the energy harvester to multidirectional wind, this paper proposes a wind energy harvester to scavenge wind-induced vibration energy. The harvester comprises a cylindrical beam instead of conventional thin rectangular cantilevers, a bluff body (square prism or circle cylinder), and a piezoelectric tube bonded to the bottom side of the beam for energy conversion. Benefiting from the symmetry of the cylindrical structure, this harvester can respond to airflow from every direction of the two-dimensional plane. The performance of the harvester under a wind speed range of 1.5–8 m/s has been tested. The results demonstrate that the proposed harvester can respond to the wind from all directions of the two-dimensional plane. It provides a direction for the future in-depth study of multidirectional wind energy harvesting.

## 1. Introduction

Wind-induced vibration is widespread in nature. In recent years, numerous types of research have focused on energy harvesting from wind-induced vibration. The conversion methods of vibration energy to electrical energy include piezoelectric transduction [1,2] and electromagnetic transduction [3,4]. The harvester uses vortex-induced vibration (VIV) [5,6], flutter [7,8], galloping [9,10], and wake galloping [11,12] of structures to scavenge kinetic energy from wind. In addition, it has great application prospects in the field of wireless sensor networks [13].

To enhance the performance of wind-based vibration energy collectors, methods such as optimizing the shape of the blunt body [14,15], hybrid energy harvesting [16,17], introducing nonlinearity [18,19], and optimizing interface circuits [20,21] have been reported. In addition, some researchers have proposed to enhance the efficiency of energy harvesting through multidirectional energy harvesting. For example, Lin et al. [22] reported a 3D-vibration energy collector by using magnetoelectric transducers. It showed a bandwidth of 3.2 Hz and a maximum power density of 44.98 μW/cm^3^. Xu et al. [23] designed a three-dimensional piezoelectric energy harvester by the internal resonance of the cantilever-pendulum system. Jiang et al. [24] developed a multidimensional vibration energy collector with a spring-mass structure, with the impact of the ball acting on piezo beams to achieve electrical output. Deng et al. [25] proposed a harvester with a double-branched beam to scavenge vibration energy. The experiments showed that the device can greatly enhance harvesting performance, both horizontally and vertically. Wu et al. [26] designed a piezo spring based on binding folders. The pendulum connected to the tip of the spring can catch vibration-based energy from the entire three-dimensional space. A new VIV-based water flow energy harvester was reported by Gong et al. [27] The collection angle range was successfully extended to 360° by adding a guide wing to the cantilever beam. Yang et al. [28] realized multidirectional vibration energy harvesting by using water as the energy capture medium.

In terms of multidirectional wind energy collection, Zhao et al. [29] utilized an arc elastic beam to pick up the wind energy and generate vibration. It can respond to wind excitation in different directions and generate different vibration forms. In another study, a cross-coupled double-beam design was proposed by Wang et al. [30], with two PZT piezoelectric plates cross-connected on the beams. Compared with the conventional wind energy harvester, this harvester has a wider effective airflow speed range. 

The above research has studied multidirectional energy harvesting, but there are few studies on multidirectional wind energy collection. Due to the randomness and instability of ambient wind energy, an investigation of multidirectional wind energy harvesting is the key to promoting the development of wind energy collectors. However, the current piezoelectric wind energy collectors mostly contain thin rectangular beams, with the transducer elements used mainly sheet structures (PZT, PVDF, MFC), which are not conducive to the response of multidirectional wind. 

This paper presents a two-dimensional, wind-based, vibration energy collector based on a cylindrical cantilever with a piezoelectric tube, which can collect two-dimensional wind flow in various directions. To our best knowledge, relevant studies on piezoelectric cylindrical beams for multidirectional wind energy collection are still rare. The cylinder-beam harvester has the advantage of miniaturization, which is conducive to integration and provides a reference for the collection and utilization of multidirectional wind energy. Experiments show that this device can effectively scavenge wind energy in multidirections and within a broad range of airflow speeds.

## 2. Structural Design of Wind Energy Harvesting

**Figure 1:**
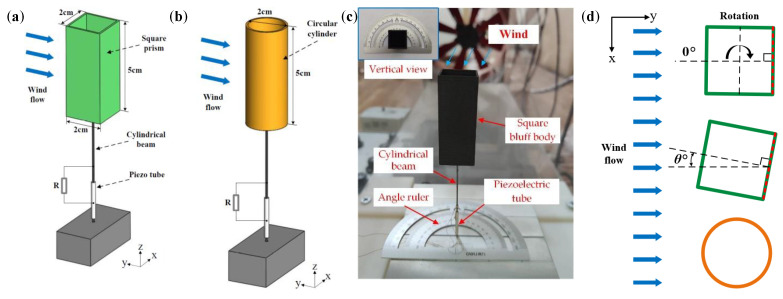
(**a**) Schematics of the two-dimensional omnidirectional harvester with a square prism and (**b**) circular cylinder. (**c**) The harvester with a square prism in the wind tunnel and (**d**) the rotation diagram of the system.

**Figure 2:**
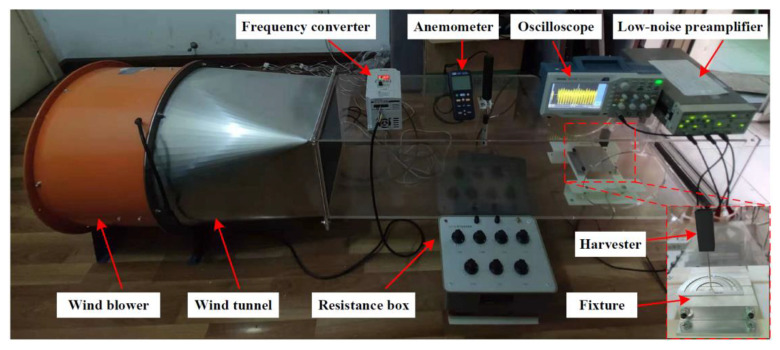
Wind tunnel experimental system.

The harvester consists of a cylindrical beam (material: Fe–Ga alloy), which supports a blunt body at the end, and a piezoelectric tube bonded to the bottom side of the beam. Figure 1a,b show the schematic of the two-dimensional omnidirectional wind energy harvester. As demonstrated in Figure 1c, a prototype is fabricated for the experiment. In addition, an electrical resistance *R* of 2.2 MΩ, which is tested as an optimal load resistance, is connected to the piezo tube. The length and diameter of the cylindrical beam are 65 mm and 1.0 mm, respectively. It is noticed that the cylindrical beam functions as an electrical conductor of the piezoelectric tube. The piezoelectric tube (soft PZT material: PIC151, capacitance: 3 nF) has a length of 20 mm, an outer diameter of 2.2 mm, an inner diameter of 1.0 mm, and a wall thickness of 0.6 mm. It is polarized in the radial direction and operates in *d*_31_ mode. In addition, the piezoelectric charge coefficients are high (*d*_33_ = 500 × 10^−12^ C/N and *d*_31_ = −210 × 10^−12^ C/N). Furthermore, the square prism and the circular cylinder are hollow, which reduces the weight of the blunt body and increases the frequency of the design, and have the same length and cross-sectional width of 5 cm and 2 cm, respectively. The wind attack angle analysis of the structure is given in Figure 1d. The incoming airflow direction can be adjusted by rotating the cylinder-shaped beam based on a protractor fixed on the fixture. In this study, the experimental tests are conducted in a small wind tunnel, which has a square cross-section with dimensions of 32 cm × 32 cm (height × width). The experiment platform is shown in Figure 2. The wind acting on the harvester is provided by an axial fan. The airflow velocity in the tunnel is controlled by a frequency converter and measured by a thermal anemometer (TES-1341, provided by TES Electrical Electronic Corp. Taiwan, China). In addition, a digital storage oscilloscope (TBS1102B, Tektronix (China) Co., Ltd., Shanghai, China) is applied to measure and save the output voltage across the load resistance. 

## 3. Results and Discussion

**Figure 3:**
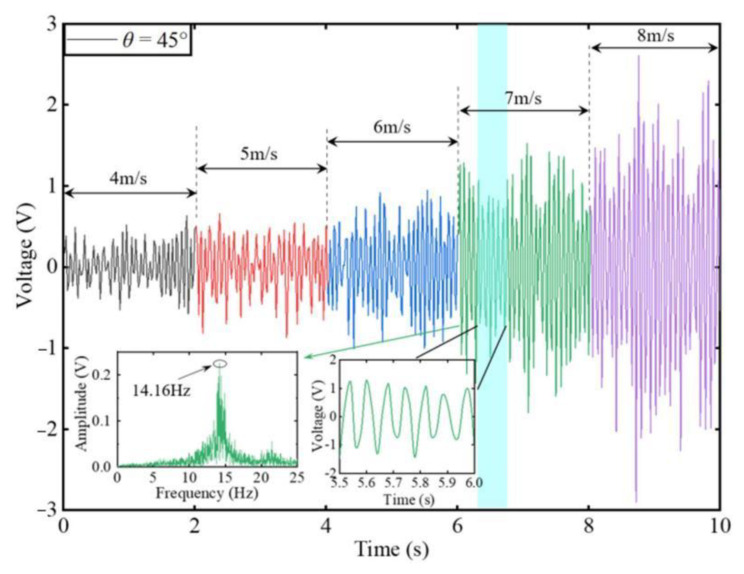
Time domain output voltage in different wind velocities for harvester with square-section cylinder when *θ* = 45°.

To characterize the output performance of the harvester with various airflow velocities, the time domain voltages are shown in Figure 3. We note that the voltage generated by the device with a square-section cylinder increases as the airflow velocity increases. However, as the wind speed increases, the unsteady airflow causes the output amplitude to turn more unstable. The oscillating frequency of the harvester is 14.16 Hz at an airflow velocity of 7 m/s. The peak-to-peak voltage of the square prism design (*θ* = 45°) can reach approximately 5.5 V when the airflow velocity reaches 8 m/s. It means the cylindrical-beam-based harvester can scavenge wind energy effectively.

**Figure 4:**
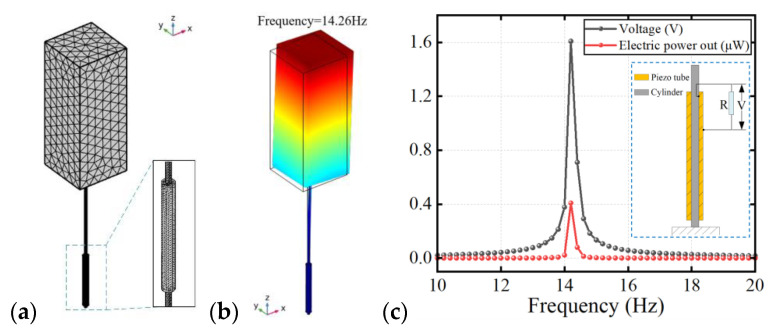
(**a**) Multiphysical field simulation model of the harvester. (**b**) Instant motion pattern at the first-order eigenfrequency of the harvester with square-section cylinder. (**c**) Voltage and electric power out versus frequency.

A three-dimensional model is established in COMSOL Multiphysics (COMSOL Co., Ltd., Stockholm, Sweden) to study the frequency response of the harvester by coupling solid mechanics, an electrostatic field, and an electrical circuit, as shown in Figure 4a. The dimensions of the simulation model and the harvester with a square-section cylinder are identical. In addition, the fixed constraint is added on the bottom side of the model. The excitation force which can be regarded as wind force is along the y-axis. A resistance is connected to the radially polarized piezo tube. The instant motion analysis of the harvester is shown in Figure 4b, with the first-order eigenfrequency of the harvester being 14.26 Hz, which is near to the oscillating frequency of the prototype. The variation of voltage and electric power out with frequency is demonstrated in Figure 4c; the mixture power can be obtained at resonance. The simulation results and the experimental tests are in good agreement.

**Figure 5:**
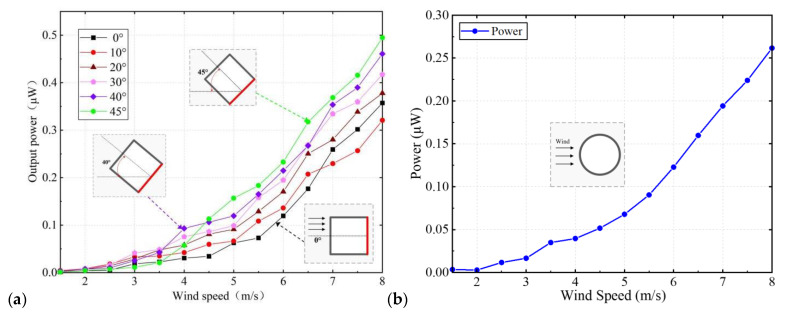
(**a**) The output power of the harvester with square-section cylinder responses to various wind velocities and wind attack angle θ. (**b**) The power output of the harvester with cylinder versus wind velocity.

We investigate the multidirectional energy harvesting capacity of the device with a square prism. When airflow with a certain velocity acts on the square prism, galloping occurs in the structure. The influence of airflow velocities (1.5 m/s–8 m/s) on the power generated by the designed harvester under different wind attack angles (0°–80°) is presented in Figure 5a. For each wind condition, measurements are repeated several times to ensure the reliability of the data. It can be seen that the power generated by the piezo tube gradually increases with airflow velocity increases, and varies with wind attack angles. Furthermore, the cut-in airflow velocity of the galloping-based collector is approximately 2 m/s. The harvester with a square prism can produce a maximum power of 0.495 μW corresponding to the RMS voltage of 1.043 V under an airflow velocity of 8 m/s. Crossflow galloping mainly occurs in cylindrical structures with non-streamlined cross-sections and corners. Thus, the harvester with a square bluff body has the largest output power at a 45-degree angle.

A prototype with a cylindrical bluff body to scavenge energy from VIV at various speeds is tested. Due to the symmetry of the cylinder, the harvester shows the same response to the airflow from all directions of the two-dimensional plane. It is apparent that the generated power increases with the airflow velocity increasing from 2 m/s to 8 m/s, as shown in Figure 5b. The harvester begins to oscillate near an airflow velocity of approximately 2.5 m/s, and a power of 0.0116 μW can be produced. The device with a cylindrical bluff body could generate power as high as 0.262 μW when the wind velocity is 8 m/s.

**Figure 6:**
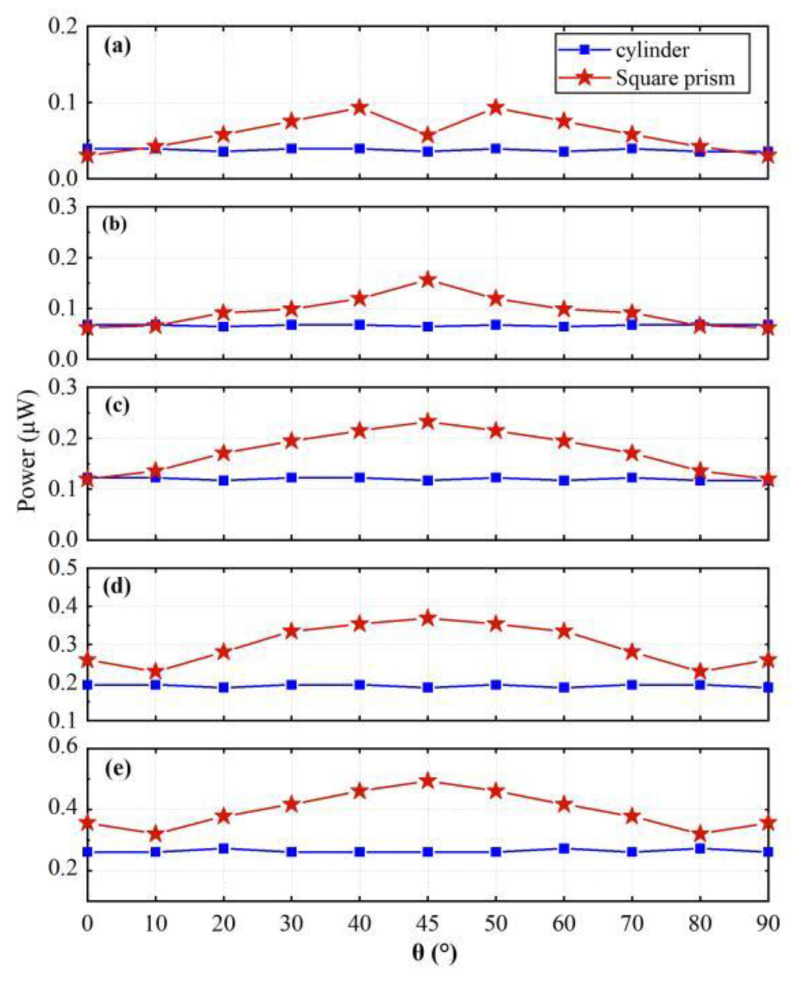
The output power of the harvesters versus angle *θ* for different wind speeds. (**a**) Wind speed: 4 m/s. (**b**) Wind speed: 5 m/s. (**c**) Wind speed: 6 m/s. (**d**) Wind speed: 7 m/s. (**e**) Wind speed: 8 m/s.

By comparing the power output of the galloping-based and VIV-based wind energy harvester at different wind velocities presented in Figure 6a–e, it can be found that the circular cylinder has a more stable output and changes more continuously, while the square prism can achieve higher power. At a low wind speed, the VIV-based harvester has better performance; with the increase of the airflow velocity, the galloping-based harvester can generate more electricity than VIV-type in all directions and is recommended in a high-wind-speed environment.

**Figure 7:**
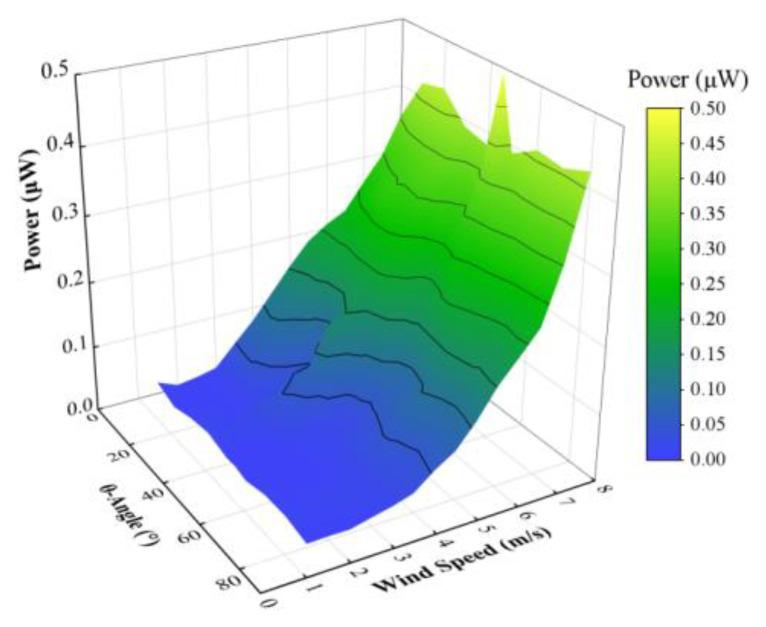
Energy harvesting capability of the harvester with a square-section cylinder.

Based on data obtained from experiments, we have Figure 7, which shows the energy harvesting capability of the harvester with a square-section cylinder. It shows that the wind attack angle will slightly influence the output power. The plotted curve surface in Figure 7 has changed slightly in the wind attack angle direction, with the overall trend to increase along the wind speed direction, which is consistent with Figure 5. 

**Figure 8:**
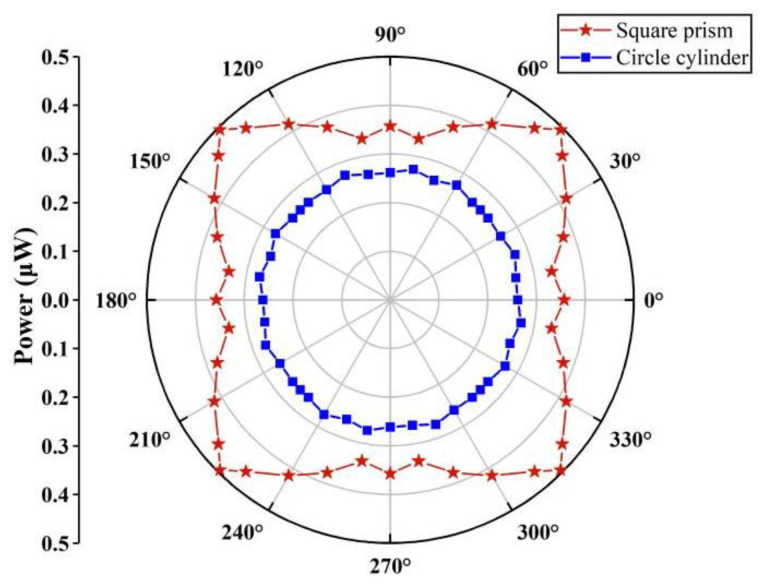
Power responses of the harvester with square prism and circle cylinder at a wind speed of 8 m/s.

Figure 8 shows the power responses of the harvesters with square-section cylinders and circle cylinders under a wind speed of 8 m/s. Results indicate that the cylinder-beam-based harvester can overcome the wind directional sensitivity and obtain power out in each direction. The galloping-based harvester has better performance than the VIV-based harvester at a high airflow velocity. The circle cylinder harvester can generate approximately 0.262 μW of power in each direction. The power harvested by the square prism collector fluctuates with the wind directions (*θ*). The maximum (0.495 μW) and minimum power (0.353 μW) are achieved when the angle of attack is 45° and 0°, respectively.

## 4. Summary

In summary, we have designed a two-dimensional, omnidirectional, wind-induced energy harvester based on a cylindrical cantilever, a piezoelectric tube, and a bluff body. The square prism or cylinder bluff body is fixed at the end of a long straight cylindrical piezoelectric cantilever. Due to the structural advantages of the cylindrical cantilever beam, the proposed harvester can respond well to wind energy from all directions. The characteristics of the harvester with a square prism or a cylinder have been studied comparatively. The results show that the harvester performs well in the direction-varying airflow environment. The harvester with a square prism can generate more power than a circular cylinder at high wind speeds. Furthermore, the power of 0.353–0.495 μW can be obtained at a wind speed of 8 m/s in each direction. The harvester with a cylinder bluff body mainly produces vortex-induced vibration, while the harvester with a square bluff body produces cross-flow galloping motion. The proposed cylinder-beam-based wind energy harvester could provide some useful guidance for future research of multidirectional wind-based vibration energy collection, and promote the application of wind energy harvesters in MEMS and wireless sensor networks.

## Data Availability

The data presented in this study are available on request from the corresponding author.
